# Sarcome fibromyxoïde de localisation inhabituelle

**DOI:** 10.11604/pamj.2015.21.92.7078

**Published:** 2015-06-05

**Authors:** Hind Benhiba, Badredine Hassam

**Affiliations:** 1 Hind Benhiba, Service de Dermatologie, CHU Ibn Sina, Université Mohamed V, Rabat, Maroc

**Keywords:** Sarcome, tissus mous, tumeur du dos du pied, Sarcoma, soft tissue, tumor of the back of the foot

## Image en medicine

Le sarcome fibromyxoïde de bas grade est une tumeur rare des tissus mous. Nous rapportons l'observation d'un patient de 54 ans consultant pour une tumeur du dos du pied droit évoluant depuis 2 ans. L'examen dermatologique notait la présence d'une tumeur bourgeonnante sous cutanée prenant la quasi-totalité du dos du pied droit, d'environ 10 cm de grand axe, douloureuse à la palpation et saignant au moindre contact. Le reste de l'examen clinique était sans particularités. L’étude histologique et immuno-histochimique d'une biopsie cutanée objectivait un processus tumoral d'allure sarcomateuse à cellules fusiformes en faveur d'un sarcome fibromyxoïde de bas grade anti-Vimentine+ anti CD34+ anti PS100- anti EMA- anti-Bcl2-. Le patient a été adressé en chirurgie et a bénéficié d'une amputation de l'avant pied de type Charcot. Le sarcome fibromyxoïde de bas grade a été décrit pour la première fois en 1987 par Evans. Moins de 150 cas sont décrits dans la littérature. Il s'agit d'une tumeur sous-diagnostiquée, souvent confondue avec d'autres entités, principalement localisée dans les tissus mous profonds des régions proximales des membres et du tronc. Histologiquement, cette variété se caractérise par l'alternance de zones myxoïdes et fibreuses constituées de cellules fusiformes. L'immunohistichimie est primordiale pour confirmer le diagnostic. Cette tumeur a un potentiel de récidive locale et de métastases même après plusieurs années, d'où l'intérêt d'un suivi à long terme. Notre observation clinique est particulière par la rareté du sarcome fibromyxoïde de bas grade ainsi que par sa localisation inhabituelle distale au niveau du pied.

**Figure 1:**
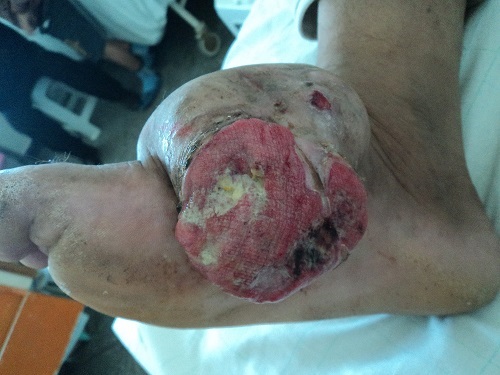
Tumeur sous cutanée du pied droit

